# Supplementary figures and images for: Prolonged inflammation leads to ongoing damage after spinal cord injury
Source: PLoS One. 2020 Mar 19;15(3):e0226584. doi: 10.1371/journal.pone.0226584 (PMC7081990; doi:10.1371/journal.pone.0226584)

## Slide 1
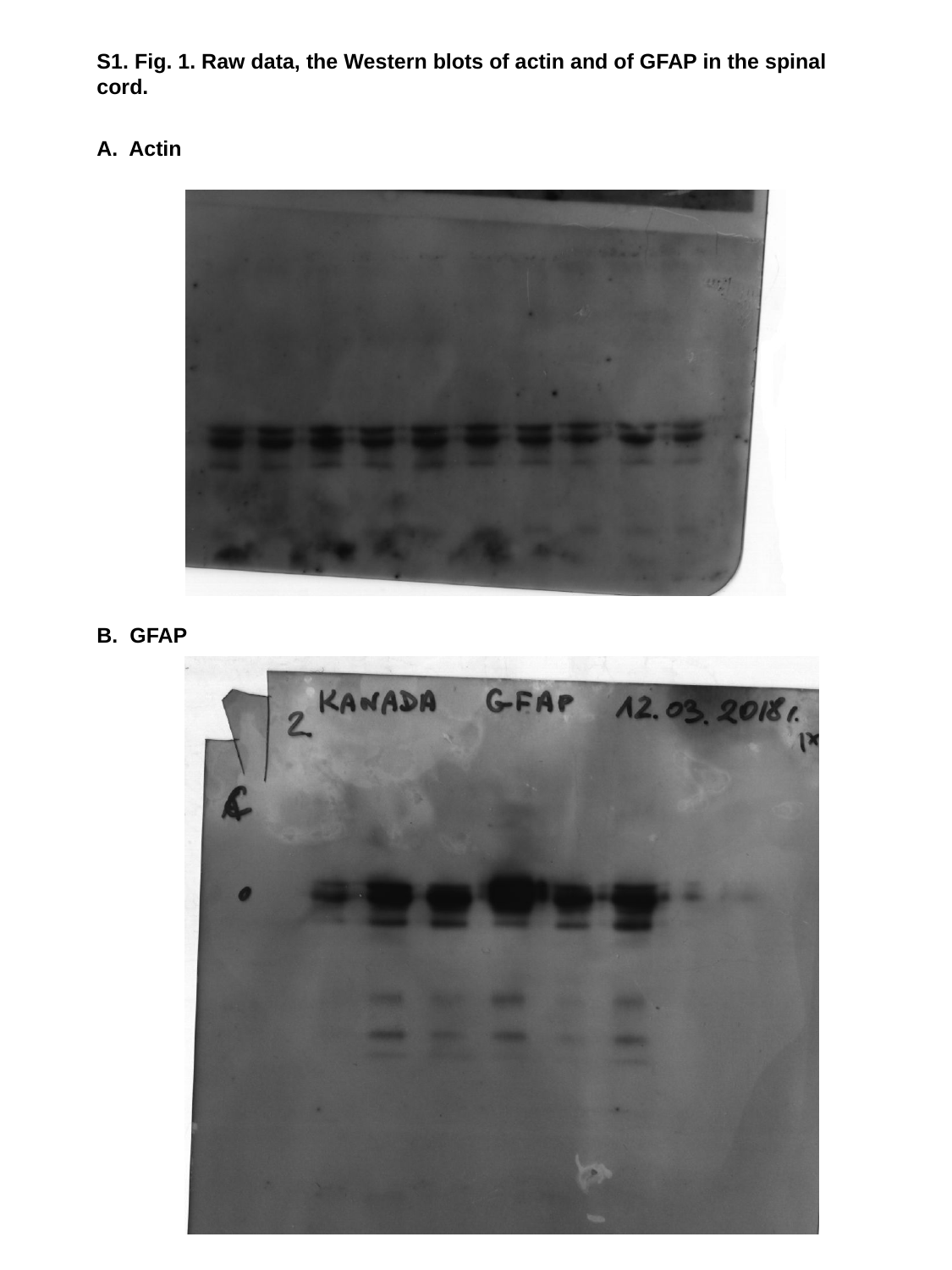

S1. Fig. 1. Raw data, the Western blots of actin and of GFAP in the spinal cord.
A. Actin
B. GFAP

Supplement: S1 Fig — A raw data, the Western blots demonstrating bands of actin in the protein extract of the spinal cord of intact LE and LES rats and of SCI rats at 2, 7, 28 and 112 days post-injury. B, Raw data, the Western blots demonstrating bands of glial fibrillary acidic protein (GFAP) in the protein extract of the spinal cord of intact LE and LES rats and of SCI rats at 2, 7, 28 and 112 days post-injury. (PPTX) [file pone.0226584.s001.pptx]
